# dbPSP 2.0, an updated database of protein phosphorylation sites in prokaryotes

**DOI:** 10.1038/s41597-020-0506-7

**Published:** 2020-05-29

**Authors:** Ying Shi, Ying Zhang, Shaofeng Lin, Chenwei Wang, Jiaqi Zhou, Di Peng, Yu Xue

**Affiliations:** Key Laboratory of Molecular Biophysics of Ministry of Education, Hubei Bioinformatics and Molecular Imaging Key Laboratory, Center for Artificial Intelligence Biology, College of Life Science and Technology, Huazhong University of Science and Technology, Wuhan, Hubei 430074 China

**Keywords:** Sequence annotation, Protein databases

## Abstract

In prokaryotes, protein phosphorylation plays a critical role in regulating a broad spectrum of biological processes and occurs mainly on various amino acids, including serine (S), threonine (T), tyrosine (Y), arginine (R), aspartic acid (D), histidine (H) and cysteine (C) residues of protein substrates. Through literature curation and public database integration, here we reported an updated database of phosphorylation sites (p-sites) in prokaryotes (dbPSP 2.0) that contains 19,296 experimentally identified p-sites in 8,586 proteins from 200 prokaryotic organisms, which belong to 12 phyla of two kingdoms, bacteria and archaea. To carefully annotate these phosphoproteins and p-sites, we integrated the knowledge from 88 publicly available resources that covers 9 aspects, namely, taxonomy annotation, genome annotation, function annotation, transcriptional regulation, sequence and structure information, family and domain annotation, interaction, orthologous information and biological pathway. In contrast to version 1.0 (~30 MB), dbPSP 2.0 contains ~9 GB of data, with a 300-fold increased volume. We anticipate that dbPSP 2.0 can serve as a useful data resource for further investigating phosphorylation events in prokaryotes. dbPSP 2.0 is free for all users to access at: http://dbpsp.biocuckoo.cn.

## Introduction

As one of the most well-characterized and important post-translational modifications (PTMs), protein phosphorylation plays an essential role in almost all signalling pathways and biological processes, from eukaryotes to prokaryotes^1–5^. This reversibly dynamic process is precisely modulated by protein kinases (PKs) and protein phosphatases (PPs), which are involved in linking or removing a phosphate group at specific residues of protein substrates^1–5^. The first eukaryotic phosphoprotein was discovered in 1883 by Olof Hammarsten, a Swedish biochemist, who detected phosphorous in a secreted protein, casein, from milk^6^. Although later studies demonstrated that many proteins can be phosphorylated in eukaryotes, it was long debated whether protein phosphorylation also exists in prokaryotes until the discovery of isocitrate dehydrogenase in *Escherichia coli*, the first identified prokaryotic phosphoprotein, in 1979^7,8^. In contrast with eukaryotic phosphorylation, which occurs mainly at specific serine (S), threonine (T) and tyrosine (Y) residues of proteins^5^, prokaryotic protein phosphorylation can occur at additional types of amino acids, such as arginine (R), aspartic acid (D), histidine (H) and cysteine (C)^1,9–13^. Given the importance of phosphorylation in the regulation of protein functions^11–13^, the identification of novel phosphorylation sites (p-sites) in proteins is fundamental for understanding the molecular mechanism and regulatory roles of prokaryotic phosphorylation.

Previously, experimental identification of p-sites with conventional biochemical assays was usually labour intensive, time consuming and expensive and was accomplished in a low-throughput (LTP) manner. The LTP methods mainly included site-directed mutagenesis (SDM) of candidate p-sites^14^, *in vitro* kinase assay (IKA) to identify potential kinase-specific p-sites^15^, detection of p-sites in purified proteins with LTP mass spectrometry (LTP-MS)^16^, and N-terminal sequencing of phosphopeptides (NSP)^17^. The quality of p-sites identified in LTP studies is higher, because usually multiple assays were performed, and the biological functions of p-sites were also carefully analyzed. Recently, advances in the development of proteomic techniques using high-throughput MS (HTP-MS) have enabled the large-scale phosphoproteomic identification of p-sites in prokaryotic proteins^18–21^. For example, Macek *et al*. conducted phosphoproteomic profiling to detect 54 phosphoserine (pS), 16 phosphothreonine (pT) and 8 phosphotyrosine (pY) residues of 78 proteins in *Bacillus subtilis*, as well as 81 pS/pT/pY sites of 79 *E. coli* phosphoproteins^18,19^. For arginine phosphorylation, Elsholz *et al*. systematically identified 121 phosphoarginine (pR) residues in 87 *B. subtilis* proteins^20^, whereas Schmidt *et al*. later quantitatively characterized 134 phosphoproteins with 217 pR sites in *B. subtilis*^21^. More recently, Lai *et al*. detected 159 phosphohistidine (pH) and 69 phosphoaspartic acid (pD) sites of 197 phosphopeptides in nine prokaryotic organisms^13^. Because an increasing number of LTP and HTP p-site investigations have been reported, the collection, curation, integration and annotation of known phosphoproteins and p-sites in prokaryotes will provide invaluable information for better understanding the host-pathogen interaction and development of antimicrobial agents.

**Fig. 1:**
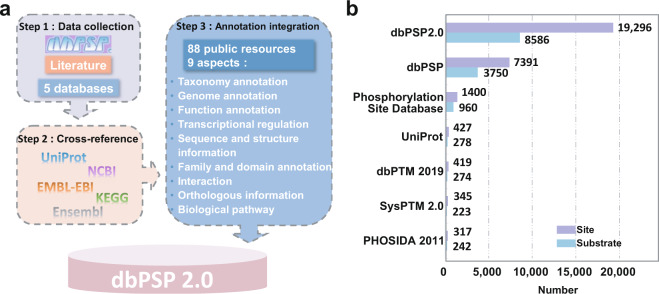
An overview of the dbPSP 2.0 database. (**a**) Flowchart for creating the database. First, we manually re-curated all entries in version 1.0 to ensure the data quality, searched PubMed to find newly identified p-sites, and integrated known p-sites from other public databases. Then, we mapped all phosphoproteins to public data sources for cross-referencing. In addition to basic information, we further integrated various annotations from 88 public databases that covered 9 aspects: (*i*) taxonomy annotation, (*ii*) genome annotation, (*iii*) function annotation, (*iv*) transcriptional regulation, (*v*) sequence and structure information, (*vi*) family and domain annotation, (*vii*) interaction, (*viii*) orthologous information, and (*ix*) biological pathway. (**b**) A comparison of the numbers of prokaryotic p-sites in dbPSP 2.0 and in other databases.

In 2015, we developed a new database of phosphorylation sites in prokaryotes (dbPSP) 1.0, which contained 7,391 experimentally identified p-sites, including 2,709 pS, 2,174 pT, 2,187 pY, 142 pR, 84 pD, 90 pH and 5 phosphocysteine (pC) sites, in 3,750 phosphoproteins of 96 prokaryotes^22^. Compared with the second largest resource, the Phosphorylation Site Database, which curated approximately 1,400 prokaryotic p-sites^23^, dbPSP 1.0 had a > 4-fold greater data volume. At that time, few annotations were provided, except limited information on p-sites. Due to the large number of prokaryotic p-sites found in recent studies, here we created dbPSP 2.0, which contains 19,296 known p-sites in 8,586 proteins from 200 prokaryotic organisms, through literature curation and public database integration (Fig. 1a, Supplementary Table 1). Furthermore, we carefully annotated these phosphoproteins and p-sites through integrating the knowledge from 88 publicly accessible databases, covering 9 aspects. In contrast with dbPSP 1.0 (~30 MB), this updated database possesses ~9 GB of data, with a 300-fold increased volume. We confirmed that dbPSP 2.0 will be continuously updated and can provide a much more useful resource for exploring protein phosphorylation in prokaryotes.

## Results

### dbPSP update

#### Entries of newly reported p-sites

Compared with version 1.0, version 2.0 contains 11,905 new entries (Fig. 1b). Through literature curation and public database integration, dbPSP 2.0 contains 19,296 non-redundant p-sites on seven different types of amino acid residues in 8,586 substrates from 200 prokaryotic species (Supplementary Table 1). In our dataset, there are 18,576 and 671 p-sites derived from HTP and LTP studies, respectively. The derivation of 96.27% known p-sites from HTP studies indicated the importance and usefulness of MS-based phosphoproteomic profiling for studying prokaryotic phosphorylation. In addition to version 1.0, we also compared dbPSP 2.0 to other existing databases, including the Phosphorylation Site Database^23^, UniProt^24^, dbPTM 2019^25^, SysPTM 2.0^26^ and PHOSIDA^27^, and our database contained a much higher number of known phosphoproteins and p-sites in prokaryotes (Fig. 1b). For each p-site, its corresponding gene name, UniProt accession number, organism, phylum, phosphorylated position, residue type, flanking peptide, data type, experimental method and original reference(s) have been present (Supplementary Table 1).

#### Distribution of phosphoproteins and p-sites for different residue types and different phyla

**Fig. 2:**
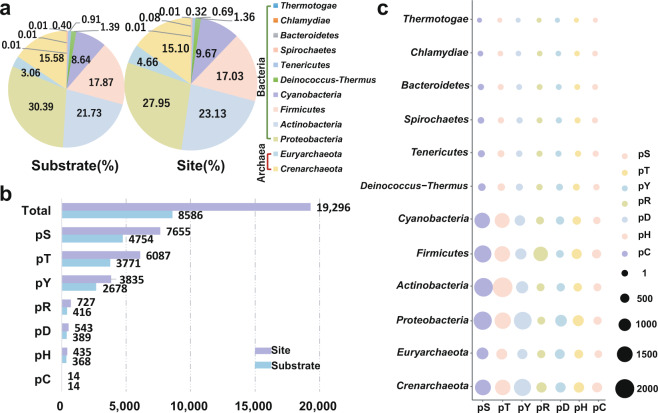
The distribution of phosphoproteins and p-sites for different phyla and different residue types in prokaryotes. (**a**) The distribution of phosphoproteins and p-sites in various phyla. (**b**) The numbers of different types of phosphoproteins and p-sites in dbPSP 2.0. (**c**) The distribution of different residue types among different phyla.

In dbPSP 1.0, known p-sites were taken from 96 prokaryotic organisms belonging to 11 phyla, *Crenarchaeota*, *Euryarchaeota*, *Proteobacteria*, *Actinobacteria*, *Firmicutes*, *Cyanobacteria*, *Deinococcus-Thermus*, *Tenericutes*, *Spirochaetes*, *Chlamydiae* and *Thermotogae*^22^. Due to the new data accumulation, known p-sites have been extended to 200 prokaryotic species in 12 phyla by adding a new phylum, *Bacteroidetes* (Fig. 2a). The distribution of numbers of p-sites among different phyla was analyzed, and it was observed that more p-sites were identified in *Proteobacteria* and *Actinobacteria* than in other phyla, with proportions of 27.95% and 23.13%, respectively (Fig. 2a). The *Proteobacteria* phylum comprises a number of extensively studied microorganisms, such as the most widely used model organism *E. coli* in microbiological studies^7,8^, and a human pathogen *Shigella flexneri* that causes bacillary dysentery mainly in children and results in 14,000 deaths per year^28^. In *Actinobacteria* phylum, one of the most notorious species is *Mycobacterium tuberculosis*, which is the causative agent of tuberculosis (TB) and annually causes 1.5 million deaths^29^. Due to the high virulence of *M. tuberculosis*, two related species including the slow-growing *Mycobacterium bovis*^30^ and the fast-growing *Mycobacterium smegmatis*^30^ were established as models to study mycobacterial physiology. Additionally, we analyzed the distribution of p-sites on different types of amino acid residues and found that pS, pT and pY sites appear more frequently than other types of residues and occupy proportions of 39.67%, 31.55% and 19.87%, respectively (Fig. 2b). Moreover, the distribution of different types of p-sites among the 12 phyla was evaluated (Fig. 2c). The most pR sites were detected in *Firmicutes*, whereas *Proteobacteria* had the highest number of pD and pH sites (Fig. 2c). Additional detailed data statistics can be viewed at http://dbpsp.biocuckoo.cn/Statistics.php.

### Coverage of phosphoproteins in different species

**Fig. 3:**
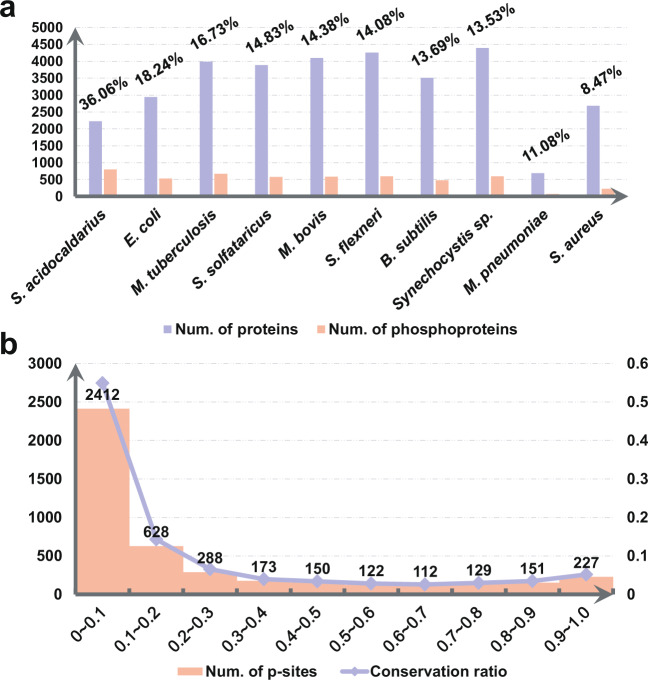
The coverage of phosphoproteins and the conservation of p-sites. (**a**) The coverage values of phosphoproteins in top 10 prokaryotes. (**b**) The distribution of the conservative ratio of p-sites in MSA results.

Due to data limitation, here we only calculated the coverage values of phosphoproteins in 50 species with ≥10 phosphorylated substrates (Supplementary Table 2). For each prokaryote, its proteome set was downloaded from UniProt^24^ by searching the corresponding Proteome ID, e.g., UP000001018 for *Sulfolobus acidocaldarius* (strain ATCC 33909/DSM 639/JCM 8929/NBRC 15157/NCIMB 11770) (https://www.uniprot.org/proteomes/?query=taxonomy:330779). Then the proportion of phosphoproteins against all protein products were counted, and top 10 species with higher coverage values were shown. From the results, we found that the coverage values of the 10 prokaryotes ranged from 8.47% (*Staphylococcus aureus*) to 36.06% (*S. acidocaldarius*) (Fig. 3a). Previously, it was estimated that about 30% of human proteins might be phosphorylated^31^, and a later study demonstrated that at least 75% of human proteins are phosphorylated *in vivo*^32^. Thus, when more and more phosphoproteomic studies are performed for prokaryotes, the coverage values of their phosphoproteins will be undoubtedly increased.

### New annotations

#### Multiple-layer annotation of prokaryotic phosphoproteins

For convenience, dbPSP 2.0 was organized as a phosphoprotein-centred database. To provide an integrative annotation of known phosphoproteins and p-sites, we provided a variety of cross-references to public data sources. For example, gene and protein names were taken mainly from UniProt^24^, whereas corresponding accession numbers were integrated from UniProt^24^, Ensembl^33^, EMBL^34^, KEGG^35^ and NCBI GenBank^36^. Moreover, functional descriptions, protein/nucleotide sequences, and keywords were derived from UniProt^24^ to provide the basic information for each phosphoprotein entry, while the primary references with PMIDs were provided for each p-site. The gene ontology (GO) annotations in the Gene Ontology resource^37^ were also included if available. Furthermore, the knowledge from 88 additional public resources, such as ChEMBL^38^, BacDive^39^, PDB^40^, IUPred2A^41^, InterPro^42^, BioGRID^43^, EggNOG 5.0^44^ and Reactome^45^, was integrated to comprehensively annotate the prokaryotic phosphoproteins. These resources covered 9 aspects, namely, taxonomy annotation, genome annotation, function annotation, transcriptional regulation, sequence and structure information, family and domain annotation, interaction, orthologous information and biological pathway (Fig. 1a). A brief summary of all public resources integrated in dbPSP 2.0 can be accessed at: http://dbpsp.biocuckoo.cn/Links.php. For these resources, the annotation datasets can be downloaded at http://dbpsp.biocuckoo.cn/Download.php.

#### Dynamic 3D structure details for phosphoproteins

For each phosphoprotein with available 3D structures characterized by X-ray crystallography or NMR spectroscopy, a representative 3D structure was selected for intuitive visualization. Users can select all or specific p-sites for visualizing their locations on protein structures.

#### HTP p-site classification

In phosphoproteomic studies, phosphopeptides were derived from mass spectrometry spectral datasets, usually with a false discovery rate (FDR) of 0.01 at the peptide-spectrum match (PSM), peptide and protein level for quality control. To pinpoint an exact p-site in a phosphopeptide, a localization probability (LP) score could be calculated by a variety of tools, such as MaxQuant^46^. LP scores range from 0 to 1, and a higher LP score represents a higher probability of a detected site being a real p-site. Since HTP p-sites were identified from different studies with different confidence, we classified all collected HTP p-sites into four classes based on their LP scores if available, namely, class I (LP > 0.75), class II (LP ≤ 0.75 and >0.5), class III (LP ≤ 0.5 and ≥0.25), and class IV (LP < 0.25), as previously described^46^. In most of these HTP studies, different reference databases, distinct search engines and/or diverse parameter configurations were adopted for phosphopeptide detection in different organisms. Thus, the aggregation of false positive identifications might result in a considerable higher FPR value in the cumulative dataset. A re-analysis of all raw MS datasets under a unified platform will generate phosphopeptides with much higher quality, although such an effort is not within the scope of dbPSP 2.0, which directly collected known p-sites from published literature.

#### Multi-alignment (MSA) of orthologs

Here, potential orthologues of known phosphoproteins were obtained from Clusters of Orthologous Groups of proteins (COG)^47^. For each orthologous group, all protein sequences were multi-aligned using MUSCLE^48^, and a conservation ratio was calculated for the sequences containing the same types of phosphorylatable residues against all sequences in the group. The distribution of the conservation ratio ranged from 0 to 1 was illustrated for all p-sites in the orthologous groups (Fig. 3b), and we only detected 227 p-sites with a conservation ratio > 0.9 (Supplementary Table 3). These highly conserved p-sites might be useful for the investigation of conserved functions of phosphorylation in prokaryotes.

### Browse lists and detailed phosphoprotein information page

**Fig. 4:**
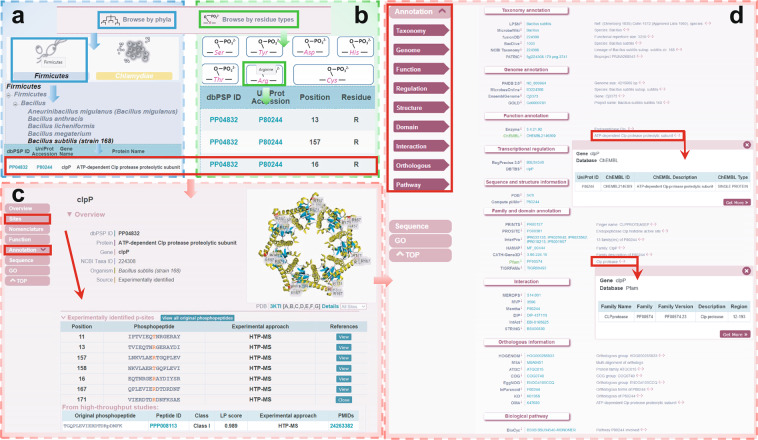
The browse options of the dbPSP 2.0 database. (**a**) Browse by phyla option. (**b**) Browse by residue types option. (**c**) Detailed information for ClpP, with known p-sites, in *B. subtilis* (strain 168). (**d**) Detailed annotations for ClpP.

dbPSP 2.0 was developed with a user-friendly website interface, and multiple browse and search options were implemented to conveniently query the data. Here, we chose *B. subtilis* ClpP, an ATP-dependent Clp protease proteolytic subunit, as an example to introduce the usage of dbPSP 2.0. Two browse options, ‘Browse by phyla’ (Fig. 4a) and ‘Browse by residue types’ (Fig. 4b), are accessible to browse the data. In the option ‘Browse by phyla’, 12 representative diagrams for all phyla are listed. The user can click the phylum to link the taxonomic category of the given phylum (Fig. 4a). The user can select ‘*Bacillus subtilis* (strain 168)’ to retrieve a list of phosphoproteins in a tabular format with ‘dbPSP ID’, ‘UniProt Accession’, ‘Gene Name’, ‘Protein Name’ and ‘Organism’ (Fig. 4a). In the option ‘Browse by residue types’, the user can choose one of the 7 residue types to browse all phosphoproteins with the given phosphorylation residue type. For example, by clicking the diagram of arginine, all proteins with pR sites are listed (Fig. 4b). Through selecting ‘PP04832’, the dbPSP ID of ClpP (Fig. 4a,b), the detailed phosphoprotein page for ClpP, is displayed (Fig. 4c,d). For a brief overview, the dbPSP ID, protein/gene names, organism, and dynamic structure details are presented (Fig. 4c). The ‘Sites’ part provides mainly detailed information on p-sites, and the original peptide and primary reference can be shown by clicking the ‘View’ button of each p-site (Fig. 4c). To access additional information on the phosphoprotein, users can click the label ‘Annotation’ on the left menu and select the interesting aspect to access the corresponding resources (Fig. 4d). For each resource, the annotation details are presented on a new page after clicking the ‘More’ icon (Fig. 4d). In addition to the browse options, multiple search options, including ‘Substrate Search’, ‘Peptide Search’, ‘Advanced Search’, ‘Batch Search’ and ‘BLAST Search’, were also developed for users to easily access the database.

### Sequence preferences of different types of p-sites

**Fig. 5:**
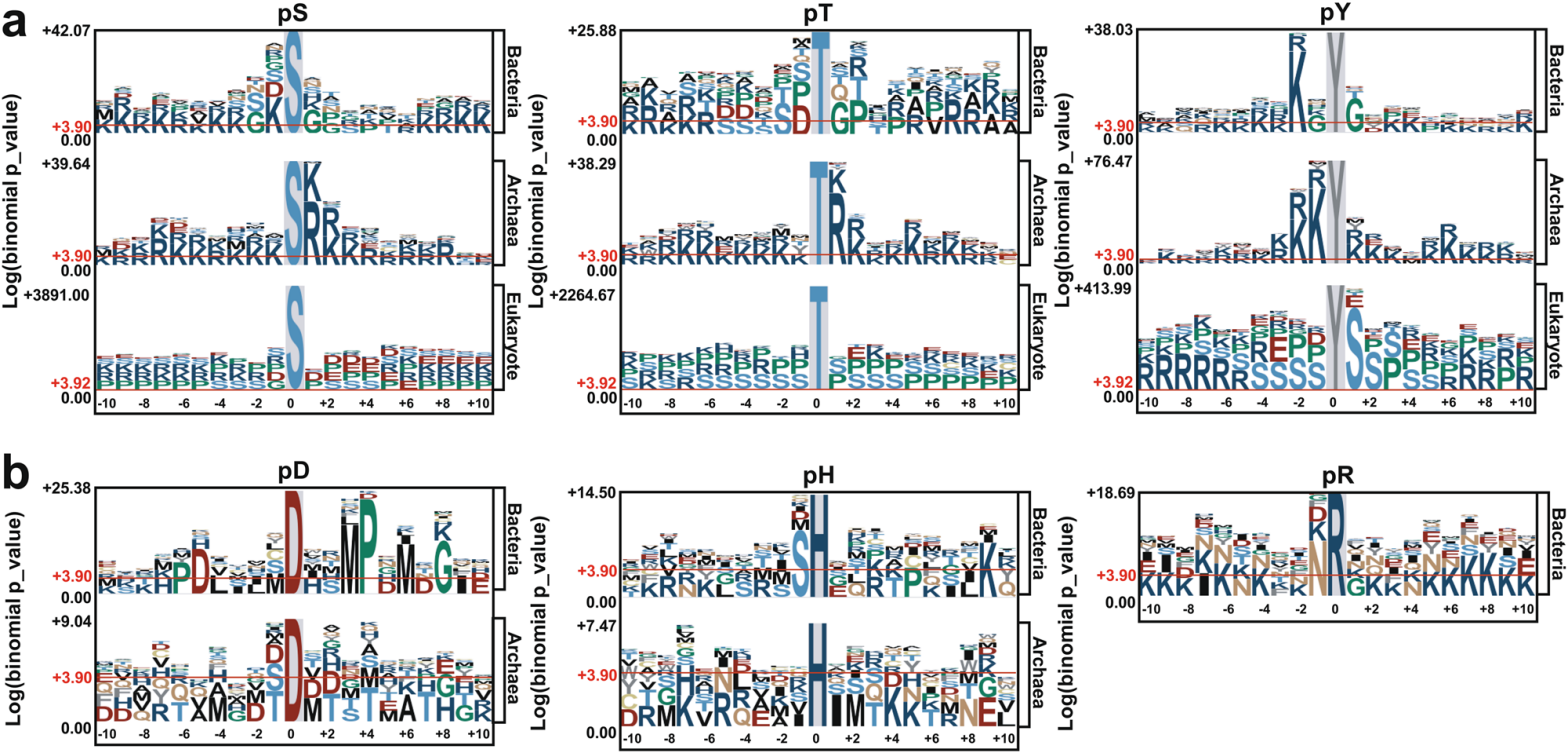
Analyses of sequence preferences for p-sites in prokaryotes with pLogo^49^. (**a**) For pS, pT and pY residues, comparisons of sequence preferences among bacteria, archaea and eukaryotes are shown. (**b**) The sequence preferences of pD, pH and pR sites in bacteria and archaea. Due to data limitations, pR sites in only bacteria were analyzed.

Due to the limited number of pC sites, here we only analyzed the sequence preferences of pS, pT, pY, pR, pD and pH sites by using pLogo^49^ for bacteria and archaea (Fig. 5). For prokaryotic pS, pT and pY sites, we also compared their sequence preferences to those of eukaryotic phosphorylation, including 382,105 pS, 123,247 pT and 59,824 pY sites by integrating two previously developed databases, dbPAF^50^ and dbPPT^51^. For pS and pT sites in archaea, R or lysine (K) residues most frequently occur at the +1 position, with a lesser extent at the +2 position (Fig. 5a). In bacteria, K residues are over-represented at the −1 position for pS sites, whereas S, D, glycine (G) and proline (P) are enriched at the −2, −1, +1 and +2 positions for pT sites, respectively (Fig. 5a). For pY sites, S residues frequently appear at the +1 position for eukaryotic phosphorylation, whereas K residues preferentially appear at the −2 position for bacteria and the −1 and −2 positions for archaea (Fig. 5a). For prokaryotic pD sites, methionine (M) and P residues are over-represented at the +3 and +4 positions around p-sites in bacteria but not archaea (Fig. 5b). For pH sites, S residues preferentially appear at the −1 position for bacteria (Fig. 5b). Due to data limitation, the sequence preference of pR sites in only bacteria was analyzed, and asparagine (N) residues are enriched at the −1 position (Fig. 5b).

### Application of dbPSP

After the publication of dbPSP 1.0, it has been visited more than 180,000 times and has served as a highly useful resource for studying prokaryotic phosphorylation^50,52–56^. For example, Garcia-Garcia *et al*. re-analyzed the phosphoproteomic datasets in dbPSP and found that phosphoproteins are essential for the regulation of the cell cycle and DNA-mediated processes in bacteria^52^. With the help of dbPSP, Venkat *et al*. experimentally validated that phosphorylation of S280 decreases the enzyme activity of malate dehydrogenase (MDH) in *E. coli*^53^. Additionally, Lin *et al*. utilized p-site information in dbPSP to analyze phosphoproteomic data and dissected the dynamic alteration of phosphorylation in various phosphoproteins during antibiotic treatment and resistance^54^. Moreover, Hasan *et al*. adopted pS and pT sites in dbPSP as training datasets and developed a useful tool, Microbial Phosphorylation Site predictor (MPSite), for predicting microbial p-sites^55^. In addition, the phosphorylation data of representative prokaryotes from dbPSP was utilized for kinase motif enrichment analysis, and the results demonstrated that most eukaryotic phosphorylation motifs could not be recovered in prokaryotes^56^.

In dbPSP 2.0, we collected and curated newly identified p-sites in prokaryotic phosphoproteins, which could present more complete information on phosphorylation in prokaryotes. Furthermore, dbPSP 2.0 has rich annotations for phosphoproteins and p-sites, which is critical for exploring the function and mechanism of phosphorylation events. In addition, the MSA results of orthologues were provided in this database and will be important for discovering conserved functional p-sites in prokaryote cells. Based on previous studies, dbPSP could work as a well-curated data resource of prokaryotic phosphoproteins to provide helpful support for phosphoproteomic analysis, tool development, and the investigation of prokaryotic phosphorylation events. We anticipate that the updated dbPSP 2.0 could be a comprehensive data resource for better understanding the importance of protein phosphorylation in prokaryotes.

## Discussion

Protein phosphorylation is one of most well-studied PTMs and is reported to be involved in regulating numerous cellular processes in prokaryotic cells^8,57^. In 2015, we collected 7,391 known p-sites of 3,750 proteins in 96 prokaryotes from published literature and developed dbPSP 1.0^22^ to contain these datasets. Due to the accumulation of phosphorylation information, here we released dbPSP 2.0 by adding 11,905 new entries to include newly discovered phosphoproteins and p-sites in prokaryotes. Furthermore, the rich annotations derived from 88 public databases were integrated. In total, dbPSP 2.0 contained 19,296 known p-sites in 8,586 phosphoproteins and occupied the size of ~9 GB, with a 300-fold increase compared to that of version 1.0.

**Fig. 6:**
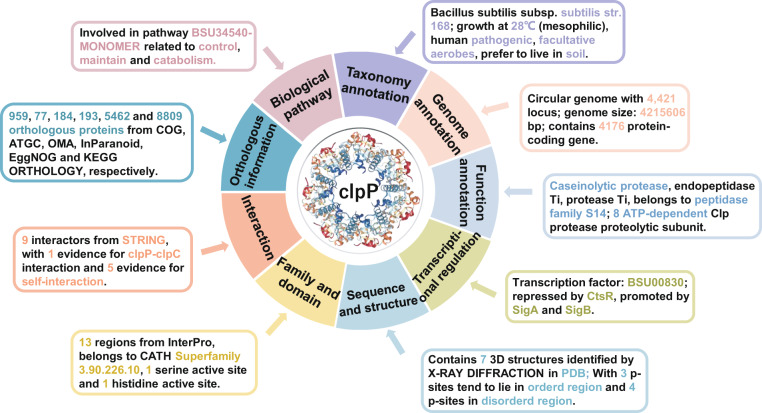
An overview of multiple-layer annotations for *B. subtilis* ClpP in dbPSP 2.0.

In this study, to cover the diverse biological roles of prokaryotic phosphoproteins, we included multiple-layer knowledge from other databases to comprehensively annotate phosphoproteins. For example, the prokaryotic ClpP enzyme plays an important role in modulating various biological processes, such as cellular stress response, pathogenesis and homeostasis^58^. Inhibiting the function of ClpP was reported to affect the infectivity and virulence of microbial pathogens^59^. Moreover, the arginine phosphorylation of ClpP was essential for maintaining its function^20,21,60^. As shown in Fig. 6, the *B. subtilis* protease ClpP is annotated as a serine peptidase and participates in eliminating damaged proteins during heat shock, and its activity can be repressed by CtsR as well as by 20,697 compounds. Meanwhile, ClpP might interact with 9 partners and self-assemble in hexameric ring structures (Fig. 6). In particular, we found nearly 15,700 records from 6 orthologous databases to demonstrate that ClpP is a highly conserved subunit in prokaryotes, and the results are consistent with previous studies. In addition, the functional domain and p-site information of ClpP were also provided. In dbPSP 2.0, the curated data resources of p-sites and phosphoproteins as well as annotation information are downloadable at http://dbpsp.biocuckoo.cn/Download.php.

In summary, the dbPSP 2.0 database will be continuously maintained and updated when new p-sites in prokaryotes are identified. In addition to adding additional annotations from other public databases, we will further develop computational tools for the prediction of prokaryotic p-sites. We anticipate that this database can provide helpful support for better understanding the regulatory mechanisms and functions of phosphorylation in prokaryotes.

## Methods

### Data collection and update

In dbPSP 1.0, we manually collected 7,391 p-sites in 3,750 non-redundant prokaryotic phosphoproteins from the literature^22^. In this study, the phosphorylation events in prokaryotes newly reported since 2014 were considered and collected. To obtain known p-sites from the literature, we searched the PubMed database with multiple general keywords, such as ‘bacteria phosphoproteomics’, ‘archaea phosphorylation’, ‘archaebacteria phospho-site’. All the retrieved 39,997 articles were manually curated to collect the experimentally identified prokaryotic p-sites, and collected p-sites were then mapped to protein sequences obtained from UniProt (release 2019_05)^24^ (Fig. 1a). We also integrated the prokaryotic p-sites from other public databases, with 1,400, 427, 419, 345 and 317 p-sites from Phosphorylation Site Database^23^, UniProt^24^, dbPTM 2019^25^, SysPTM 2.0^26^ and PHOSIDA^27^, respectively (Fig. 1b). These datasets were cross-checked with our manually collected dataset and then integrated into the dbPSP 2.0 database.

### Structure data collection and prediction

The 3D structures of phosphoproteins for intuitive visualization were obtained from the PDB^40^ if available. A JavaScript molecular visualization library, 3Dmol.js^61^, was used to support the dynamic structure chart in the browser interface. In addition, the probabilities of disordered binding regions and disorder propensity values were predicted by using ANCHOR2^41^ and IUPred2^41^, respectively. The details are provided on the phosphoprotein page.

### Web interface construction

HTML, PHP and JavaScript were applied to develop the web interface as the front-end. The MySQL server was applied to manage the data as the back-end. The backlog and cache data will be cleared regularly, and the dbPSP database will be maintained and optimized continuously.

## Supplementary information

Supplementary Table 1

Supplementary Table 2

Supplementary Table 3

## Data Availability

All the collected phosphoproteins, p-sites and various annotations are freely available at http://dbpsp.biocuckoo.cn/Download.php. For convenience, phosphorylation datasets can be downloaded in three data types, including the total dataset, the phylum-specific datasets, and the residue-specific datasets The datasets of phosphoproteins in prokaryotes have been uploaded to figshare^62^, 10.6084/m9.figshare.11436879. The annotation datasets were classified by their functional categories, and users can choose the corresponding options based on their own purposes. All data sets in dbPSP are made available under a Creative Commons CC 3.0 BY license (https://creativecommons.org/licenses/by/3.0/cn/).

## Code availability

The source code of dbPSP 2.0 database has been uploaded to GitHub: https://github.com/BioCUCKOO/dbPSP2.0.
